# Supplementary material for: Trophic Position of the Species and Site Trophic State Affect Diet Niche and Individual Specialization: From Apex Predator to Herbivore
Source: Biology (Basel). 2023 Aug 10;12(8):1113. doi: 10.3390/biology12081113 (PMC10452534; doi:10.3390/biology12081113)
Supplement: Supplementary file 1 [file biology-12-01113-s001.zip › 02 Vejrik_S1 Supplementary Materials.pdf]

Supplementary Materials for

Trophic position of the species and site trophic state affect diet niche and individual specialization: from apex predator to herbivore

Lukáš Vejřík *et al*.

This PDF file includes:

S1: Calculation and results of isotopic half-lives in four tissues of the species studied

Figure S1: Mean isotopic half-lives in all tissues (plasma, blood, fin, muscle) for all species studied (rudd, Eurasian perch, Northern pike, and European catfish)

Calculation and results of isotopic half-lives in four tissues of the species studied

Calculation of isotopic half-life

Isotopic half-life in all four tissues (fin, muscle, blood, plasma) was calculated separately for each species at each site. Carbon isotopic half-life can be predicted according to Stephen & Crowther [52]:

$$\log_{10} (^{13}\mathrm{C}\ t_{½}) = 1.6668 + 0.1935 * \log_{10} \mathrm{BM} + -0.0153*\mathrm{BT}$$

where $^{13}\mathrm{C}t_{1/2}$ is the half-life of δ^{13}C (in days), BM is an organism's body mass (g), and BT is its body temperature (°C).

Similarly, nitrogen isotopic half-life can be estimated using the equation:

$$\log_{10} (^{15}\mathrm{N}t_{½}) = 1.6884 + 0.1933 * \log_{10} \mathrm{BM} + -0.0149*\mathrm{BT}$$

where $^{15}\mathrm{N}t_{1/2}$ is the half-life of δ^{15}N (in days).

Body mass was the mean mass of each species at each site. Temperature, for where the species occurs, was obtained from data loggers located in the entire depth profile at all sites. The mean depth of occurrence of each species was determined based on acoustic telemetry tracking of individual species at the site (see [66] for description of the acoustic telemetry and temperature measurements). Mean temperature of species occurrence for fin, blood, and plasma, was obtained from a period of 20–40 days (according to species body mass) prior to sample collection. For muscle, the period was 80–150 days. The time was determined from the isotopic half-life of the tissues, the entire turnover corresponds to 4–5 times the half-life, and the process has a logarithmic character [64].

Results of isotopic half-lives in four tissues of the species studied

Isotopic half-lives differed significantly among species due to differences in body mass and temperature of their occurrence. It also differed to a lesser extent among sites. European catfish with mean mass of 8.3 kg (±5.18; SD) and Northern pike with a mean mass 3.75 kg (±SD: 3.06) had the longest and the second longest half-lives, respectively. Rudd and Eurasian perch with mean mass of 0.44 (±0.25; SD) and 0.39 kg (±0.25, SD), respectively, had shorter half-lives than European catfish and Northern pike, and the half-lives of the corresponding tissues were similar (Fig. S1).

The isotopic half-life in plasma of rudd was the shortest, ranging from 15.5 to 17.3 days, depending on the site. In contrast, the half-life in plasma of European catfish (species with the longest tissue turnover) ranged from 32.7 to 38.8 days. The mean isotopic half-life in fin and blood was 26 and 30 days, respectively, for rudd and Eurasian perch at all sites. For predatory fish, the mean half-life in blood was 39 days for both Northern pike and European catfish. The mean half-lives in fins were 41 and 44 days for Northern pike and European catfish, respectively. The greatest differences in mean half-lives were in muscles, with the shortest in Eurasian perch (78 days) and the longest in European catfish (153 days). The results are summarized in Figure S1.

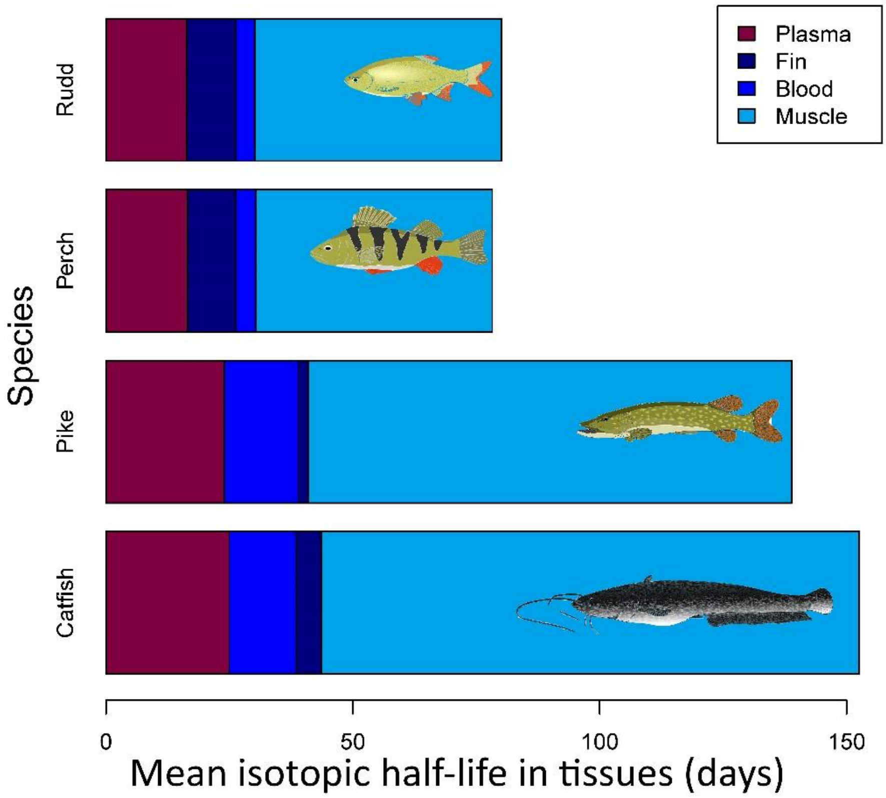

Figure S1. Mean isotopic half-lives in all tissues (plasma, blood, fin, muscle) for all species studied (rudd, Eurasian perch, Northern pike, and European catfish).
